# Microfluidic cantilever detects bacteria and measures their susceptibility to antibiotics in small confined volumes

**DOI:** 10.1038/ncomms12947

**Published:** 2016-10-04

**Authors:** Hashem Etayash, M. F. Khan, Kamaljit Kaur, Thomas Thundat

**Affiliations:** 1 Faculty of Pharmacy and Pharmaceutical Sciences, University of Alberta, Edmonton, Alberta, Canada T6G 2E1; 2 Department of Chemical and Materials Engineering, University of Alberta, Edmonton, Alberta, Canada T6G 2V4; 3 Chapman University School of Pharmacy, Harry and Diane Rinker Health Science Campus, Chapman University, Irvine, California 92618-1908, USA

## Abstract

In the fight against drug-resistant bacteria, accurate and high-throughput detection is essential. Here, a bimaterial microcantilever with an embedded microfluidic channel with internal surfaces chemically or physically functionalized with receptors selectively captures the bacteria passing through the channel. Bacterial adsorption inside the cantilever results in changes in the resonance frequency (mass) and cantilever deflection (adsorption stress). The excitation of trapped bacteria using infrared radiation (IR) causes the cantilever to deflect in proportion to the infrared absorption of the bacteria, providing a nanomechanical infrared spectrum for selective identification. We demonstrate the *in situ* detection and discrimination of *Listeria monocytogenes* at a concentration of single cell per μl. Trapped *Escherichia coli* in the microchannel shows a distinct nanomechanical response when exposed to antibiotics. This approach, which combines enrichment with three different modes of detection, can serve as a platform for the development of a portable, high-throughput device for use in the real-time detection of bacteria and their response to antibiotics.

## Abstract (web summary)

Analysis of bacteria and their response to antibiotics in real time is challenging. Here the authors report a microcantilever based system that can detect and discriminate between bacteria species and, due to the ability to discriminate between alive and dead samples, measure response to antibiotics.

## Graphical abstract

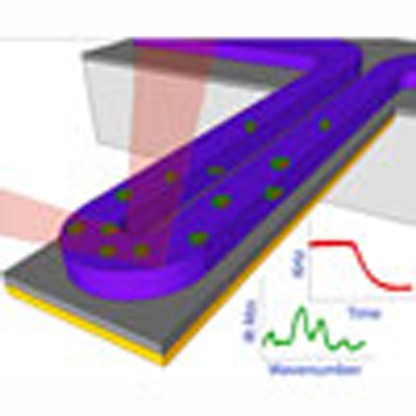

Current methods for detecting bacteria and measuring their response to antibiotics lack sensitivity, selectivity, stability and the ability for real-time analysis^1^. Laboratory-based detection methods, such as agar plates and broth dilution assays, are inconvenient and require a minimum of 24 h to complete, depending on the bacterial species^2^. Rapid detection techniques, such as antibody–antigen assays (for example, enzyme-linked immunosorbent assay)^3^, resazurin-reduction assays^4^ (for bacterial resistance), the mycobacterial growth indicator^5^ and/or polymerase chain reaction-based methodologies^6^, are very sensitive and powerful detection tools. However, they are expensive and they are unable to distinguish between living and dead species. In addition, high sensitivity and selectivity in real-time measurements in the stated techniques are still challenging^1^. Hence, inexpensive sensors for the rapid detection of bacteria and the determination of their susceptibility to antibiotics are urgently needed in order to combat the emergence of drug-resistant bacterial strains.

Recent developments in micro and nanofabrication allow the integration of multiple signal generation techniques into a single device to obtain orthogonal signals, which enhances the detection sensitivity and selectivity^7^. A number of versatile, highly sensitive sensors, based on microcantilevers for microbial detection, have been developed^8^,^9^,^10^. These sensing concepts rely on immobilizing specific receptors on the cantilever surface for selectively capturing the target bacteria and translating the binding into mechanical signals, as either cantilever deflection (static mode) or a shift in resonance frequency (dynamic mode)^10^. Despite many advances in these conventional modes of cantilever operation, a number of constraints still exist that limit their widespread application. First, sensitive measurement of the resonance frequency shift in a liquid environment has been limited by the low-quality factor (Q-factor) of the cantilever due to liquid damping^10^. However, static-mode cantilever operation based on surface stress such as that described by Longo *et al*.^11^ is not affected by the presence of liquid. Second, the response of the cantilever is often affected by liquid flow, which increases the signal-to-noise ratio. Laminar flow around the cantilever creates a potential barrier for the efficient capture of targets from the flowing solutions^12^. In addition, the small dimensions of the sensor decrease the capture cross-section, resulting in the reduced adsorption of target molecules. Therefore, the mode and volume associated with fluid delivery play a critical role in the capture rate of the target molecules^12^.

A suspended microchannel resonator, where a microfluidic channel is embedded inside a microcantilever, overcomes the limitations of liquid damping and achieves unprecedented mass resolution^13^. Since the liquid is inside the cantilever, the cantilever can be excited into resonance in a vacuum for increased mass resolution and higher reproducibility^14^,^15^. Despite its extraordinarily high mass sensitivity, this resonator still lacks selectivity in detection. Incorporating multimodal detection, by which multiple orthogonal signals can be monitored simultaneously, is a way to achieve the desired selectivity. Pre-concentrating analytes also increase the selectivity and sensitivity of detection. We have fabricated a microfluidic channel on a bimaterial cantilever (BMC) so as to obtain three orthogonal signals—adsorbed mass, adsorption stress and mid-infrared spectroscopy of the adsorbates, as shown in Fig. 1. Functionalizing the interior surfaces of the BMC with specific receptors allows the target bacteria to be selectively trapped inside the channel in a 50-picolitre volume. Adsorption of the bacteria causes changes in the cantilever resonance frequency, resulting from changes in the inertial mass of the liquid-filled cantilever. In addition, adsorption of bacteria results in the cantilever bending because of adsorption-induced surface stress, which results from the microfluidic channel being fabricated on top of the cantilever with cross-sectional asymmetry. Adsorption-induced stress originates from changes in free energy (free energy per unit area is surface stress) due to adsorption. A third orthogonal signal can also be obtained by illuminating the cantilever with infrared radiation. Absorption of specific infrared wavelengths by the adsorbed bacteria causes additional cantilever deflection because of non-radiative decay. The nanomechanical bending of the cantilever, as a function of illuminating wavelength, resembles the infrared absorption spectrum of the bacteria. Since infrared absorption spectroscopy is an established technique, incorporating this into the BMC system enables selective identification of bacterial strains and accurate discrimination between injured and intact cells. In this article, we applied the BMC sensor to enrich and detect *L. monocytogenes* in picolitre sample volumes with high sensitivity and selectivity using three orthogonal signals. In addition, the metabolic activity of the adsorbed bacteria resulted in nanometre-scale fluctuations that are larger than the Brownian motion of the cantilever. Sensitive monitoring of this fluctuation allows the sensor to discriminate between intact and dead *Escherichia coli* (*E. coli*), as well as characterize the metabolic response of *E. coli* to antibiotics.

## Results

### BMC fabrication and characterization

The BMC is fabricated using silicon nitride with a 300 nm-thick layer of gold on one side for enhanced thermal sensitivity (bi-material effect). Changes in the BMC deflection amplitude (Δ*A*) are measured using an optical-beam-deflection method, which enables recording of the resonance frequency and deflection of the cantilever simultaneously. In addition, sequential exposure to infrared radiation excites the bacteria inside the cantilever, producing heat that deflects the cantilever further. Monitoring the deflections as a function of illuminating wavelengths shows the infrared spectra of the targeted bacteria. Details of the experimental set-up, bacterial subculture and preparations, receptor immobilization, characterization and surface density studies are described in the Supplementary Figs 1–3. In addition, see Supplementary Methods for experimental details.

### Bacterial detection

To demonstrate bacterial detection, we used *L. monocytogenes*, a serious food-borne pathogen that has a mortality rate exceeding 20% (ref. 16). Before bacterial injection into the sensor (10^2^ cells in 100 μl), the inner surface of the chip was functionalized with either the anti-*L. monocytogenes* monoclonal antibody (mAb-coated BMC) or the *L. monocytogenes*-targeted antimicrobial peptide (AMP-coated BMC). In addition to its binding selectivity, the immobilized receptors on the inner BMC interface served as pre-concentrators, increasing the number of bacteria in the channel. The detailed chemistry of surface functionalization is shown in the Supplementary Fig. 1.

Figure 2 shows cantilever deflection and resonance frequency shift as a function of bacterial adsorption. Resonance frequency changes result from changes in the inertial mass caused by the immobilized receptors capturing bacteria (Fig. 2a). The mass of bacteria captured in the channel can be measured from the resonance frequency shift as 24.5 and 24. 9 ng in both AMP and mAb-coated BMC, respectively. In addition to the frequency shift, the cantilever deflection changes simultaneously as a result of the bacteria adsorption-induced surface stress, with an average differential deflection of 62±4 and 68±5 nm in both the AMP and mAb-coated BMCs (Fig. 2b). Figure 2c shows differential cantilever deflection as a function of illuminating wavelength due to infrared absorption by the bacteria trapped in the channel. The differential deflection is obtained by subtracting the infrared-heating-induced deflection of an empty cantilever from that obtained with bacterial sample loaded in the BMC. This mechanical infrared absorption of the bacteria displays a typical spectrum with a distinct absorption peak at 1,451 cm^−1^, suggesting a peptidoglycan layer of the bacterial cell wall (Fig. 2c). Absorption bands observed at 1,233 and 1,213 cm^−1^ (Fig. 2c) are due to the C–O–C ester and P=O vibrations of the bacteria phosphate diester groups, respectively. Two other vibrational bands also appear during irradiation of the sensor with higher wavelengths, indicating a P-OH (1,100 cm^−1^) and polysaccharide group (1,023 cm^−1^) in the bacterial cell wall (Supplementary Fig. 4). As reported previously, these observed infrared absorption bands are a characteristic fingerprint of the bacteria^17^,^18^,^19^,^20^.

### Sensitivity

Since sensitivity is a key determinant to the applicability of using the sensor in real applications, we conducted experiments where different concentrations of bacteria, ranging from 10^3^ to 10^6^ c.f.u. ml^−1^, were injected into the BMC. We measured the nanomechanical deflection and plotted the responses against bacterial concentrations in the samples (Fig. 2d). While the deflection signals of the control device (peptide-coated BMC) showed negligible response upon exposure to various concentrations of bacteria, the AMP- and mAb-coated BMCs showed increased bending with increased concentrations of bacteria. As the concentration of bacteria increases, so does deflection, which suggests a direct relationship to the number of bacteria bound to the functionalized surface. The results show the lowest detection limit of 100 cells per 100 μl (a single cell μl^−1^), for a signal-to-noise ratio of 3. This detection limit is clinically relevant and compares well with other reported techniques^21^,^22^,^23^,^24^. The advantages of the BMC sensor include its ability for multimodal detection with very small volumes and its enhanced sensitivity and selectivity. Other label-free devices, such as the surface plasmon resonance or quartz crystal microbalance, can only provide a single signal and are only suited for applications involving low molecular weight analytes^25^,^26^. Unlike conventional cantilevers and/or atomic force microscopy cantilevers, the BMC offers multimodal detection of liquid-phase analytes with higher selectivity, sensitivity and increased reliability.

### Selectivity

The selectivity study intended to explain the selectivity matrix as it depends on Gram-positive versus Gram-negative and the different strains of Gram-positive bacteria via elucidating the selectivity rejoinder of *L. monocytogenes* in contrast to other gram-positive strains. Figure 2e,f shows the selective detection of *L. monocytogenes*. Cantilever deflection and resonance frequency shifts for different strains in serial concentrations (Fig. 2e,f) revealed substantial discernment patterns and selective responses to *L. monocytogenes*. The differential nanomechanical cantilever deflections for different strains were clearly discernable for targeted strains (Supplementary Fig. 5a). Control experiments carried out using fluorescence microscopy verified these results (supplementary Fig. 5b). In addition, the nanomechanical infrared spectrum shows differences between bacterial species (Supplementary Fig. 6). These variations can be attributed to the asymmetric stretching of P=O in the phosphodiester backbone of nucleic acids (at ∼1,213 cm^−1^), the asymmetry of the peptidoglycan layer of the bacterial cell wall (at 1,451 cm^−1^) and the lipid groups (between 1,000 and 1,023 cm^−1^) in the bacterial cell wall. Specificity in the infrared spectra also comes from lack of interference as the flow-through approach selectively captures the targeted strains while still allowing the untargeted strains to pass through the channel. It is clear from these results that the AMP-coated BMC exhibited preferential binding towards *L. monocytogenes* relative to other strains by ∼2–3 orders of magnitude, while the mAb-coated BMC showed absolute specific response to *L. monocytogenes*, in comparison with other tested strains. We explain this differentiality towards *L. monocytogenes* by a mechanism-related behaviour of the immobilized ligands^27^,^28^. In an AMP-coated BMC, Leucocin A is a very distinctive AMP, which targets a specific membrane-bound receptor on the surface of the bacteria^29^,^30^. This receptor is more prevalent in *L. monocytogenes* than in other species. As a result, Leucocin A has a higher affinity to *L. monocytogenes* than other strains. The mAb-coated BMC targets a specific antigen on the surface of *L. monocytogenes*, which is not present in other strains. The AMP-coated BMC offers a broad-spectrum diagnostic tool by allowing the detection of pathogenic bacteria. The sensor is sufficiently stable and reusable (supplementary Fig. 7) and provides a cost-effective alternative to currently available techniques. On the downside, it is not specific for *L. monocytogenes*, as it can capture other strains with lower affinities. This can be tackled by differentiating the measured responses with respect to their strengths and flaws. In contrast, the mAb-coated BMC proposes a specific detection methodology to *L. monocytogenes* at a higher affinity rate. Although the sensor is very specific and sensitive, it cannot be used to detect multiple strains simultaneously; however, it offers a more specific device than the AMP-base (Supplementary Fig. 7).

### Antimicrobial resistance

In order to demonstrate the feasibility of using a BMC sensor to detect bacterial response to antibiotics, a small aliquot of living *E. coli* (∼10^5^ c.f.u. ml^−1^) was inserted into a BMC chip. Before insertion of the sample, the internal walls of the BMC were coated with a thin film of bacteria-adhesion molecules, (3-aminopropyl)triethoxysilane (APTES), which allowed the loose attachment of bacteria without affecting their metabolism^11^. In the first set of these experiments, the response of *E. coli* DH5α to ampicillin and kanamycin was monitored. We measured the deflection and resonance frequency shifts before and after the attachment of *E. coli*, after injecting liquid broth (LB) media, and LB-containing 10 μg ml^−1^ either, ampicillin or kanamycin (see methods and Supplementary Materials and Methods for details).

Figure 3 shows cantilever deflection and resonance frequency, as a result of *E. coli* exposure to antibiotics (ampicillin and kanamycin). The introduction of *E. coli* causes the cantilever to deflect (∼70±4.1 nm) as well as resonance frequency to shift (∼–2.6 KHz from the background). An injecting aliquot of LB media led to a slight increase in the cantilever's deflection and a decrease in the resonance frequency (+71±3 nm and –0.7 KHz). Five minutes after the injection of LB-containing ampicillin, the resonance frequency showed an increase of ∼0.2 KHz, while the deflection dropped by ∼4–5 nm. After 30 min of exposure, the resonance frequency showed a larger shift (∼+0.4 KHz, Fig. 3a), while the deflection further decreased by ∼7–9 nm (Fig. 3a). Similar to the ampicillin response, injecting kanamycin showed an increase in the resonance frequency (∼+0.1 KHz, Fig. 3b) and a drop in the deflection (∼4–6 nm) after 5 min of exposure (Fig. 3b). However, unlike ampicillin, 30 min after exposure, the resonance frequency dropped and the deflection increased compared with what was observed before the injection of kanamycin (∼+6 nm, Fig. 3b). The measured noises in the *E. coli*-immobilized BMC cantilever deflections before and after the injection of antibiotics show also significant variation between ampicillin and kanamycin (Fig. 4). It has been reported previously that changes in bacterial metabolic activity change the different stresses on the cantilever^11^. Thus, we assume that this effect may be because of metabolism-induced stress and may indicate bacterial resistance to the drugs. As can be seen in Fig. 4 upper panel, the fluctuation decreased dramatically (variance 0.65±0.053 nm^2^) compared with that observed before the ampicillin injection (variance 6.16±0.26 nm^2^). However, after the kanamycin was injected (Fig. 4 lower panel), the fluctuation was consistently higher and compared well with the observation before kanamycin injection (variance 5.61±0.046 nm^2^). In both cases, the bacterial cells seem to deactivate their metabolic processes initially after exposure to the antibiotics (short dormancy state), and then either die or recover with the addition of nutrients. Drug-induced bacteria death (ampicillin) resulted in changes in frequency, surface stress and decreased cantilever bending and fluctuation. In contrast, the bacteria exposed to kanamycin appear to have full metabolic recovery, resulting in a decrease in frequency, increase in cantilever's deflection and nanomechanical noise. Our results are in agreement with previous reports of nanomechanical noise associated with the viability of bacteria and metabolic activity^11^.

### Life versus dead bacteria

To support this hypothesis and to investigate whether bacteria have been killed or placed in a dormancy state (a period in the bacterial life cycle when physical activities are temporarily stopped in order to survive unforeseen circumstances), we removed the drugs and re-introduced LB broth media (Figs 3 and 4). As expected, the bacteria exposed to ampicillin were killed, showing a further decrease in cantilever deflection (Fig. 3a), an increase in the resonance frequency (Fig. 3a) and a decrease in the vibrational noise (Fig. 4d), compared with the bacteria exposed to kanamycin, which showed an increase in deflection (Fig. 3b), decrease in resonance frequency (Fig. 3b) and an enhanced cantilever fluctuation (Fig. 4d′). A multivariate analysis of the nanomechanical infrared spectra was carried out to determine the difference between intact and injured bacteria. Figure 5 shows the second derivative transformation analysis of the nanomechanical infrared spectra of *E. coli* placed in LB, exposed to ampicillin (Fig. 5a) or kanamycin (Fig. 5b), and then further incubated with LB after removal of the drugs. The spectra (Fig. 5a,b) showed unique infrared absorption features for the bacteria exposed to ampicillin and kanamycin. As shown in Fig. 5, the spectral data were processed by separating overlapping absorption bands and by removing baseline shifts to show the difference between intact and injured bacteria. In addition, analysing the data using the principle component analysis showed distinct clusters, corresponding to intact and injured bacteria (Fig. 5c). These results show that bacteria exposed to ampicillin have been lysed (killed), while bacteria exposed to kanamycin are alive. The distinct differences in infrared-nanomechanical spectra are arising primarily from the vibration of the molecular moieties on the bacterial cell wall (bands at 1,451 cm^−1^). Changes in the infrared spectra of bacteria during exposure to ampicillin may originate from denaturation and/or redistribution of the cell contents. It is clear that exposure to drugs such as ampicillin (which causes rupture of the cell walls or cell membranes of the bacteria) and protein re-distribution may also result in unique spectral features. These results were further confirmed using confocal microscopy imaging (Fig. 5d), which shows both live and dead bacteria (after exposure to ampicillin or kanamycin). In this experiment, the viability of the attached bacteria to the internal surface of the cantilever was evaluated by incubating the bacteria with life/dead stain for 10 min at 37 °C. The live/dead stain contained two different fluorescent dyes, which stains live cells green while staining the dead cells red because the red pigment can only adhere on damaged cell membranes. As indicated from Fig. 5d and from bacteria counting analysis, most of the *E. coli* exposed to ampicillin were killed (red stained) while 75% of the *E. coli* exposed to kanamycin were alive (green stained). The results support the conclusion that BMC readout signals, including cantilever deflection, resonance frequency shift, nano-fluctuation and the mechanical infrared-bending, are associated with the viability and metabolism of the bacteria.

To verify the connection between the nanomechanical fluctuations of the BMC and bacterial metabolism, we introduced a medium that supports bacterial metabolism, consisting of 5% glucose and collected the BMC data (Supplementary Figs 8 And 9). The drastic increase in the nanomechanical fluctuations of the cantilever clearly supports the hypothesis of increased fluctuation with an active metabolic process of the bacteria. Responses of *L. monocytogenes* and *E. coli DH5α*, confined in the BMC to Leucocin A (a ribosomally synthesized AMP of class IIa bacteriocins), were comparable to those obtained with ampicillin. These results show that a BMC can be an ideal sensor platform for testing bacterial responses to a variety of drugs (Supplementary Fig. 10).

## Discussion

The integration of photothermal infrared spectroscopy with a bimaterial microchannel cantilever—with its internal surface functionalized with receptors—overcomes the sensitivity and selectivity challenges presented by the real-time detection of bacteria and their interactions with antibiotics. By exploiting the semi-selective nature of the AMP from class IIa bacteriocins and the specific properties of mAbs, we were able to capture *L. monocytogenes* and detect it at very low concentrations, down to a single cell per μl. The BMC platform also enabled us to monitor bacterial response to antimicrobials more closely when compared with existing approaches. The detection of resistant bacteria using the nanoscale motions of living bacteria exposed to ampicillin, kanamycin and AMP is also demonstrated. In contrast to other bacterial monitoring tools, the BMC combines the selectivity of infrared spectroscopy with the thermal sensitivity of the BMC to obtain the infrared spectra of analytes in picolitres of samples. This nanomechanical infrared spectroscopy, based on calorimetry, is complementary to that of the conventional infrared spectra, which uses the Beer-Lambert law of counting photons for signal generation. However, heat-based nanomechanical spectroscopy is a direct technique for measuring infrared absorption by a sample and, since the mid-infrared is free from overtones, this wavelength range is ideal for molecular recognition. In addition, the BMC is capable of measuring the mass density of analytes with high resolution and detects analytes, including bacteria, as they pass through the cantilever's microchannel. These BMC cantilevers can be mass-produced for low cost using conventional microfabrication techniques. Capturing the target analytes inside the channel by surface immobilization enhances sensitivity as well as selectivity. Since a BMC can support multiple orthogonal signal generation concepts, the technique is highly versatile and has achieved better sensitivity, selectivity and faster responses, when compared with other approaches such as the optoplasmonic nanosensor^7^. We anticipate that these infrared-integrated BMC sensors will be useful for a wide variety of applications, ranging from food and water analysis to drug discovery and testing pharmaceutical ingredients. In the near future, it will be possible to integrate sample separation techniques with BMC platforms to achieve the full potential of the lab-on-a-chip concept.

## Methods

### BMC fabrication

**Figure 1:**
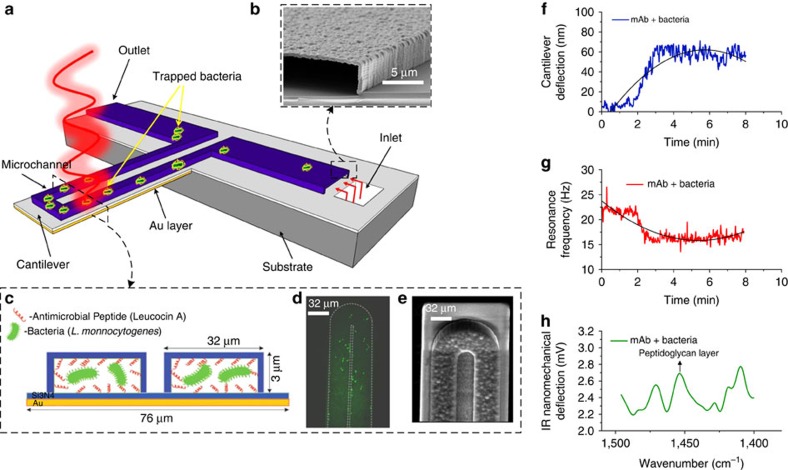
The schematic representation of the BMC and its multi-mode of operation. (**a**) BMC filled with bacteria supported on a silicon substrate. At the bottom, the BMC is coated with a 300 nm-thick layer of gold, which serves as a second element (mismatched expansion coefficients between the silicon nitride and gold layer facilitate the cantilever deflection as a localized heat is produced). The BMC was coated with a bacteria-targeted receptor and irradiated with a specific wavelength of tunable infrared light. (**b**) Scanning electron microscopy (SEM) image of the cross-section of an inlet, located on bottom side of the chip. An aqueous solution of bacteria is loaded from the inlet. (**c**) Cross-section of the 32 μm wide microchannel of the cantilever. The inner surface of the cantilever's microchannel was functionalized either with a mAb or an AMP (Leucocin A) from class IIa bacteriocins, which acted specifically against *L. monocytogenes.* (**d**) Fluorescent image from the top side of the BMC, filled with bacteria. (**e**) SEM image of the tip of the BMC. The round microchannel helps to ensure clog-free flow. (**f**) When the bacteria inside the BMC absorbs infrared light, local heat is generated that results in the nanomechanical deflection of the BMC. (**g**) The resonance frequency is sensitive to the increased mass caused by the adsorption of bacteria inside the BMC. (**h**) When the BMC is illuminated with a certain range of infrared light, a plot of the nanomechanical deflection of the BMC shows the wavelength where the bacteria absorb infrared light. This can provide excellent selectivity in a complex mixture.

**Figure 2:**
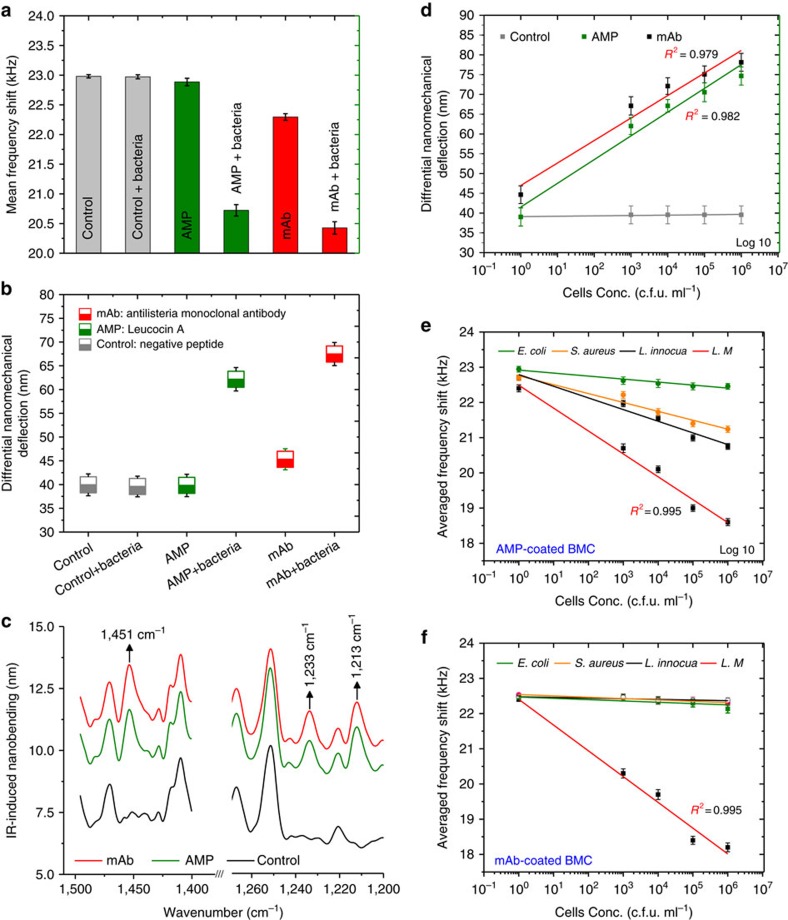
BMC multi-mode signal readout as a function of bacterial adsorption. (**a**) The mean descent in the resonance frequency shifts as a result of captured *L. monocytogenes* by either AMP or mAb-coated BMC; frequency drops as higher-density bacteria attach to the inner surface of the cantilever. In comparison with a control BMC (coated with a negative peptide), the AMP- and mAb-coated BMCs show significant responses to *L. monocytogenes* (*P*=0.032). (**b**) Nanomechanical bending of the cantilever as a result of bacteria adsorption-induced surface stress. Statistically significant deflection is observed for both AMP- and mAb-coated BMCs in comparison with the control (*P*<0.05; *n*=5). Differential deflection represents the specific binding event of the immobilized receptor to the bacteria, derived by subtracting the infrared-induced deflection. (**c**) Typical segments of BMC infrared nanomechanical spectra show the distinctive infrared absorption bands of bacteria (1,451, 1,233 and 1,213 cm^−1^). The spectra were subtracted from the background signal and smoothed 45% to decrease the noise. (**d**) Nanomechanical deflection of a BMC after exposure to serial concentrations of *L. monocytogenes* demonstrates the sensitivity of the BMC. The corresponding fit is a linear function and error bars show the corresponding s.d.'s (*n*=5). (**e**,**f**) The selectivity of the BMC towards *L. monocytogenes*; the resonance frequency of the BMC changes with the type of bacteria species tested. It shows selectivity (higher affinity to *L. monocytogenes*) with an AMP-coated BMC and specificity (capturing only *L. monocytogenes*) with a mAb-coated BMC. The data represent an average of five replicates and error bars correspond to s.d.'s.

**Figure 3:**
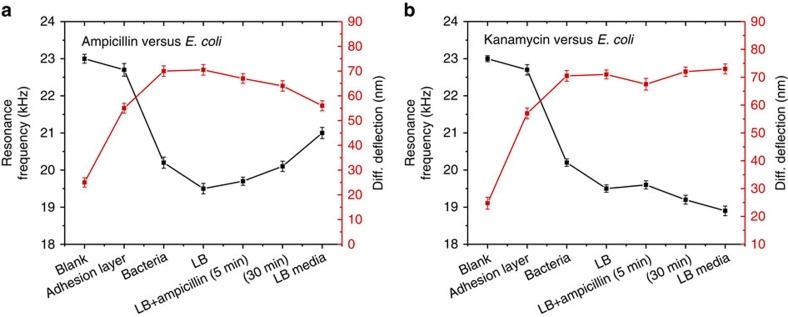
The BMC sensor displays the response of *E. coli* DH5α to antibiotics (ampicillin and kanamycin) at 0.1 μg ml^−1^. (**a**,**b**) The resonance frequency shifts and the nanomechanical deflections as a result of serial steps starting from a blank cantilever to removal of the drug and re-introduction of the LB media. A decrease in the frequency is observed with the introduction of both bacteria and LB media (**a**,**b**). Introducing ampicillin (**a**) led to an increase in the resonance frequency and a decrease in the nanomechanical deflection. Injection of kanamycin, however (**b**), led to a decrease in the resonance frequency and an increase in the nanomechanical bending. Removing antibiotics and adding LB media further confirmed that bacteria have been killed by ampicillin (no dormancy), but not by kanamycin (**b**). An average of five replicates is presented with error bars, indicating s.d.'s.

**Figure 4:**
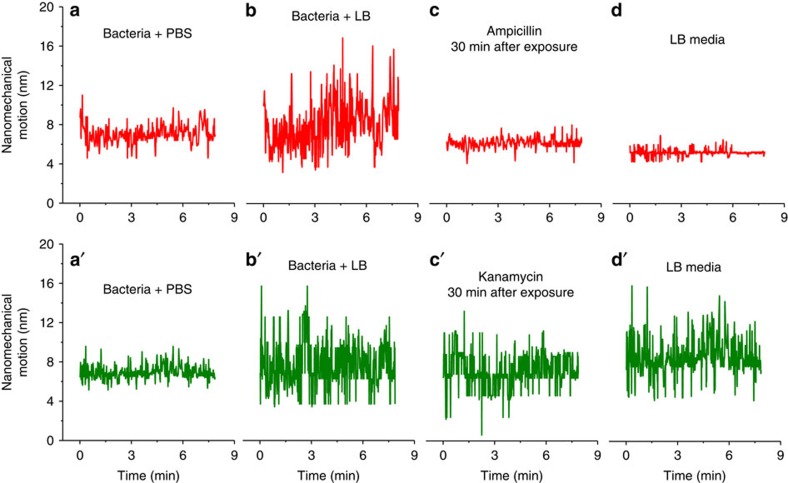
Nanomechanical fluctuation shows bacterial susceptibility to ampicillin (upper panel) and kanamycin (lower panel). (**a**,**a**′) The results of bacteria in PBS; **b**,**b**′ show the enhanced fluctuation due to the insertion of LB media into the bacteria. (**c**,**c**′) The fluctuation after exposure to antibiotics, ampicillin and kanamycin, respectively (measurement was performed 30 min after the exposure). This suggests that the *E. coli* have been killed by ampicillin but that they resist the antibacterial effect of kanamycin. Removal of the antibiotic and re-introduction of LB media to the bacteria confirmed that bacteria exposed to ampicillin have been killed (**d**), while the *E. coli* exposed to kanamycin are alive (**d**′).

**Figure 5:**
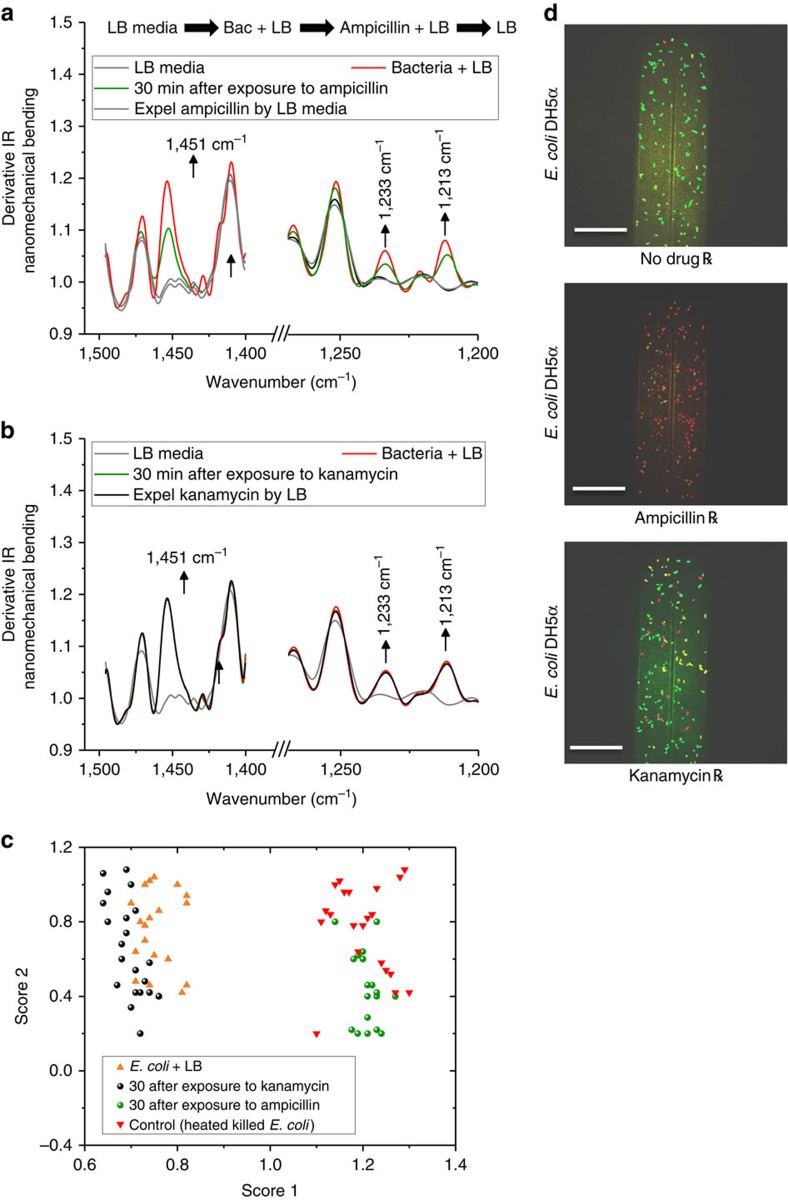
Nanomechanical infrared spectra of *E. coli*. Representative infrared second deviation analysis in the mid-infrared region for bacteria exposed to ampicillin (**a**) and kanamycin (**b**). The measurements were performed as indicated on top of the spectra, first LB media alone, followed by LB+bacteria, and the later addition of an antibiotic in LB+bacteria, and finally, exchanging the antibiotics with LB media. The infrared spectra were algorithmically preprocessed (binning, smoothing and second deviation transformation) to reduce the number of data points so as to eliminate noise. (**c**) Representation of the multivariate statistical analysis technique of principal component analysis (PCA), which selectively differentiates dead from intact bacteria after exposure to ampicillin or kanamycin, respectively. (**d**) Confocal microscopy images of the antibiotic–bacteria interaction inside the BMC were obtained (∼30 min after exposure to drugs); a live/dead viability kit was used to stain living cells green and dead cells red. Images were taken using confocal microscopy (scale bar, 22 μm).

A bi-material microcantilever (32 μm wide and 600 μm long) with a microfluidic channel (cross-section 32 μm × 3 μm) embedded on it was used in this study (Fig. 1a). The cantilevers were mircofabricated using silicon nitride and a thin layer of gold (300 nm) was deposited on one side to make them bi-material. When the bacteria inside the BMC absorb a specific wavelength of infrared, they produce localized heat, which is then transferred to the gold layer beneath the silicon nitride. Owing to a mismatch in the thermal expansion coefficients of silicon nitride and gold, the BMC deflects upwards. Changes in BMC deflection (ΔA) are measured by reflecting a laser off of the cantilever to a position-sensitive diode detector.

### Bacterial detection experiments

In the detection experiments, the microfluidic channel of the cantilever was functionalized using bacteria-targeting molecules to capture the analytes and enhance the detection sensitivity and selectivity. Two targeting molecules of *L. monocytogenes* were employed (anti-*L. monocytogenes* mAb and *Listeria*-selective AMP from class IIa bacteriocins). A peptide with nonspecific binding to *Listeria* was used as a negative control (see Supplementary Materials and Methods for further details).

In the experiment, 100 μl water samples, free from bacteria or artificially contaminated with bacteria at various concentrations (10^2^–10^5^ c.f.u. ml^−1^), were injected into the sensor and subjected to nanomechanical monitoring while the BMC were filled with liquid. Measurements of the cantilever deflection, nanomechanical infrared spectra and mass adsorption (measured as resonance frequency shifts) were taken simultaneously. As targeted bacteria pass through the narrow microfluidic channel embedded on the cantilever, they are trapped by the immobilized ligands. The bacteria absorb infrared photons at certain wavelengths and release heat to the background through the non-radiative decay process of vibrational energy relaxation. This results in a small change in the temperature of the bimetallic cantilever, causing it to bend in proportion to the quantity of the released energy. While the infrared-induced nanomechanical spectra represent the molecular signature of the bacteria inside the microchannel, the resonance frequency shifts provide real-time measurements of the specific mass of the captured bacteria. The bacteria-adsorption-induced cantilever bending was monitored at infrared wavelengths where bacteria did not absorb the infrared. Note: the interference due to infrared radiation-induced bending of the empty cantilever is eliminated by taking the differential deflection of the cantilever, which will then represent the specific binding signals of bacteria binding to the immobilized receptors. The differential deflection is obtained by subtracting the deflection of the cantilever filled with sample (bacteria) from the deflection of an empty cantilever exposed to the same infrared radiation.

### Bacterial resistance experiments

For the bacterial drug-resistance experiments, the sensor was chemically treated using a linker molecule (APTES), which provided a loose attachment of the bacteria to the surface, holding the cells in place without affecting their metabolic activity. After the treatment, the BMC was introduced into the sensor chamber to complete the analysis. Calibration of the cantilever was performed at this point by injecting a bacteria-free PBS solution and monitoring the infrared-induced nanomechanical bending, cantilevers deflection, fluctuations, as well as frequency shifts associated with the loaded materials. The measurements were used as a baseline in the analysis of subsequent experiments. The nanoscale dynamic deflection, cantilever motion, infrared absorption and the resonance frequency shifts were collected after each step. A solution containing a small aliquot of living bacteria (∼10^5^ c.f.u. ml^−1^) was introduced into the BMC and left to incubate for 10 min at ambient temperature. The BMC chamber was then rinsed with PBS to ensure removal of any floating cells that might have an impact on the results. Afterwards, a standard bacterial growth LB medium was introduced on the sensor in order to promote metabolic activities, and data were subsequently collected. The growth media was then exchanged with LB media containing antibiotics; either ampicillin or kanamycin at a concentration of 10 μg ml^−1^ and the data of resonance frequency, infrared absorption, fluctuation and cantilever nanomechanical deflections were collected twice at ∼5 and ∼30 min of exposure. The antibiotics were then removed and the LB medium was re-introduced before collecting data. To enhance the metabolism of the bacteria, we also introduced a 5% glucose solution to the bacteria after their exposure to the antibiotics. Each experiment was repeated at least five times to verify the consistency of the results, and statistical difference analysis was performed using either the unpaired *t-*test or the one-way analysis of variance test, as specified. The multivariate statistical analysis technique of principle component analysis was used for analysing the infrared data of the bacteria to differentiate injured *E. coli* from intact *E. coli*. In all statistical analyses, the significance level (*P* value) was set as 0.05. (Detailed experiments can be found in the Supplementary Materials and Methods.)

### Data avialabity

The authors declare that the some data supporting this finding are available within the article and its Supplementary Materials. Other raw data for the reported results are available from the authors upon request

## Additional information

**How to cite this article:** Etayash, H. *et al*. Microfluidic cantilever detects bacteria and measures their susceptibility to antibiotics in small confined volumes. *Nat. Commun.* **7,** 12947 doi: 10.1038/ncomms12947 (2016).

## Supplementary Material

Supplementary Information Supplementary Figures 1-10, Supplementary Methods and Supplementary References.
