# Supplementary material for: Microfluidic cantilever detects bacteria and measures their susceptibility to antibiotics in small confined volumes
Source: Nat Commun. 2016 Oct 4;7:12947. doi: 10.1038/ncomms12947 (PMC5059454; doi:10.1038/ncomms12947)
Supplement: Supplementary Information — Supplementary Figures 1-10, Supplementary Methods and Supplementary References. [file ncomms12947-s1.pdf]

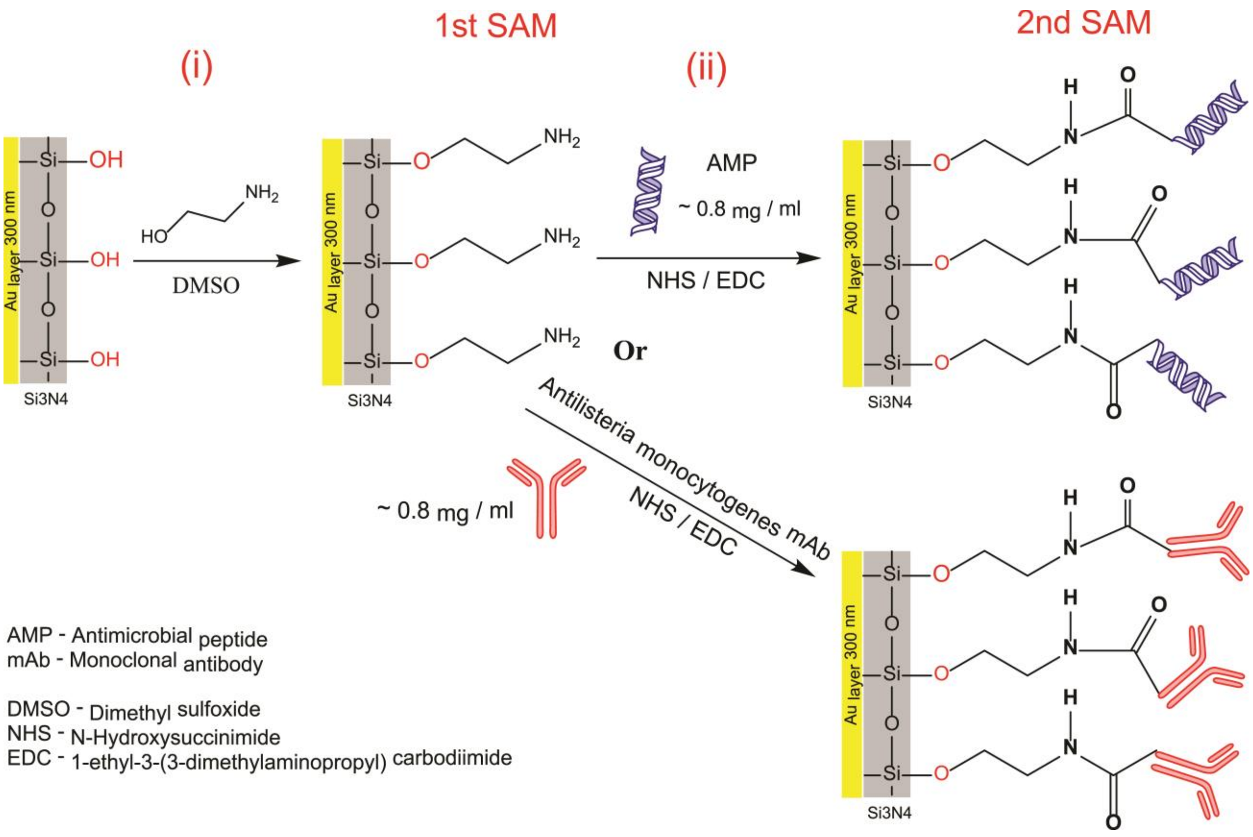

Supplementary Figure 1| BMC ligands attachment. Chemistry of the BMC surface functionalization illustrates the two steps of the AMP/ or mAb of immobilization strategy. Ethanolamine interacts with the oxidized silicon nitride surface (**i**), forming free amine groups (1st SAM) accessible for conjugation with pre-activated carboxylates of the AMP or mAb (**ii**) to custom an AMP or mAb adlayer (2nd SAM) as indicated. DMSO: Dimethylsulfoxide; NHS: N-hydroxysuccinimide; EDC: 1-Ethyl-3-(3-dimethylaminopropyl) carbodiimide.

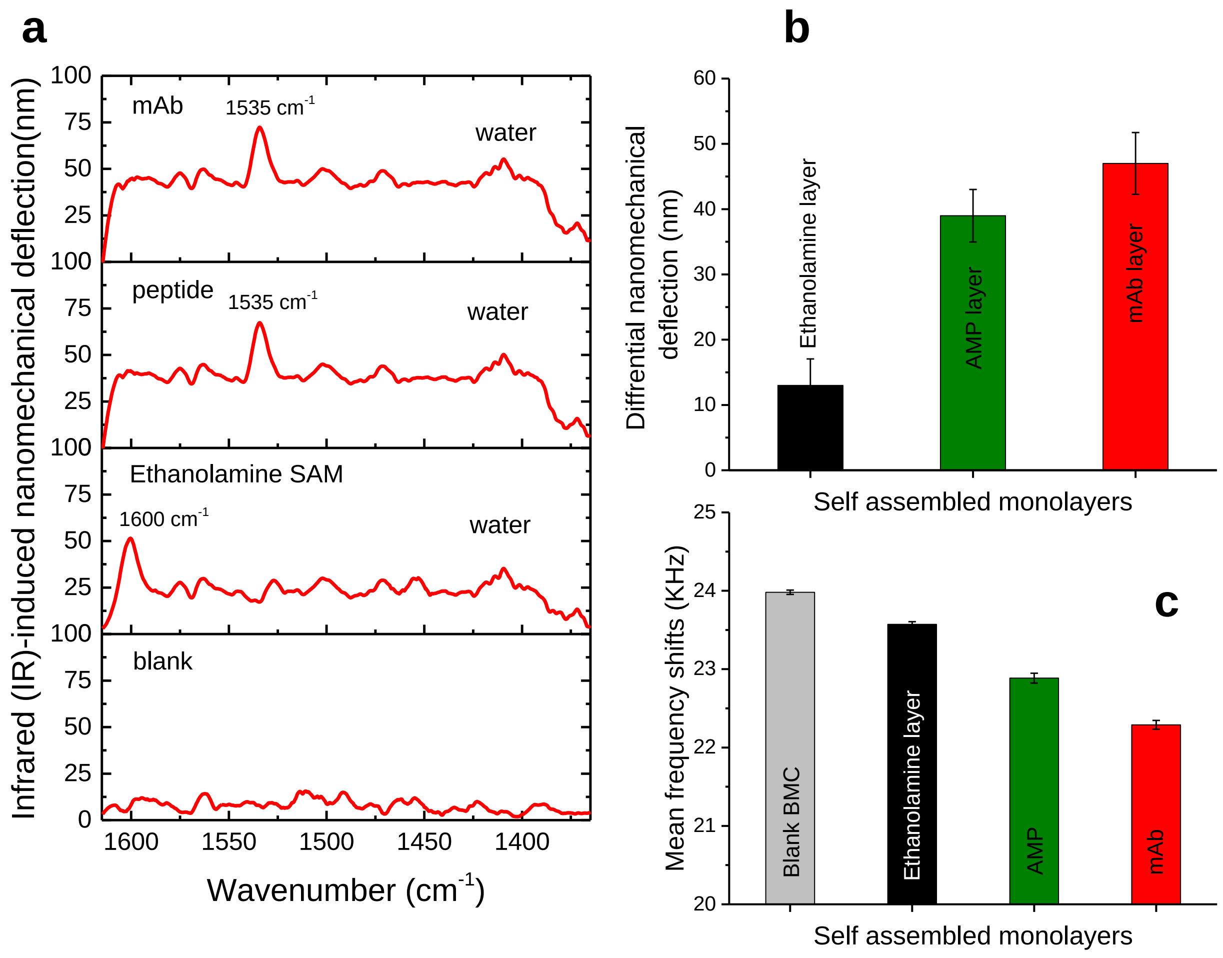

Supplementary Figure 2| Infrared signature, cantilever nanomechanical deflections and frequency shifts exhibit BMC surface functionalization. (**a**), integrated IR signals present SAM layers on the cantilever surface. Appearance of a 1ry amine band around 1600 cm^{-1} (ethanolamine panel) is an indicative signal of the ethanolamine layer-adherence. Its extinction and appearance of amide II band (1553 cm^{-1}) is a definite signal of a second SAM formation (mAb /or peptide adlayer). IR spectra were smoothed 30% and are representative of five replicates. (**b)** Differential nanomechanical deflections of the cantilever show the response of the BMC to the adhered adlayers. Averaged values are presented with error bars indicating standard deviations (n=5). (**c**) BMC resonance frequency shifts as a result of surface functionalization. Frequency drops down as higher density molecules attach to the inside shallow of the microchannel, 1st SAM (ethanolamine), AMP adlayer, mAb adlayer. Changes in the resonance frequency are measured with respect to the surface functionalization.

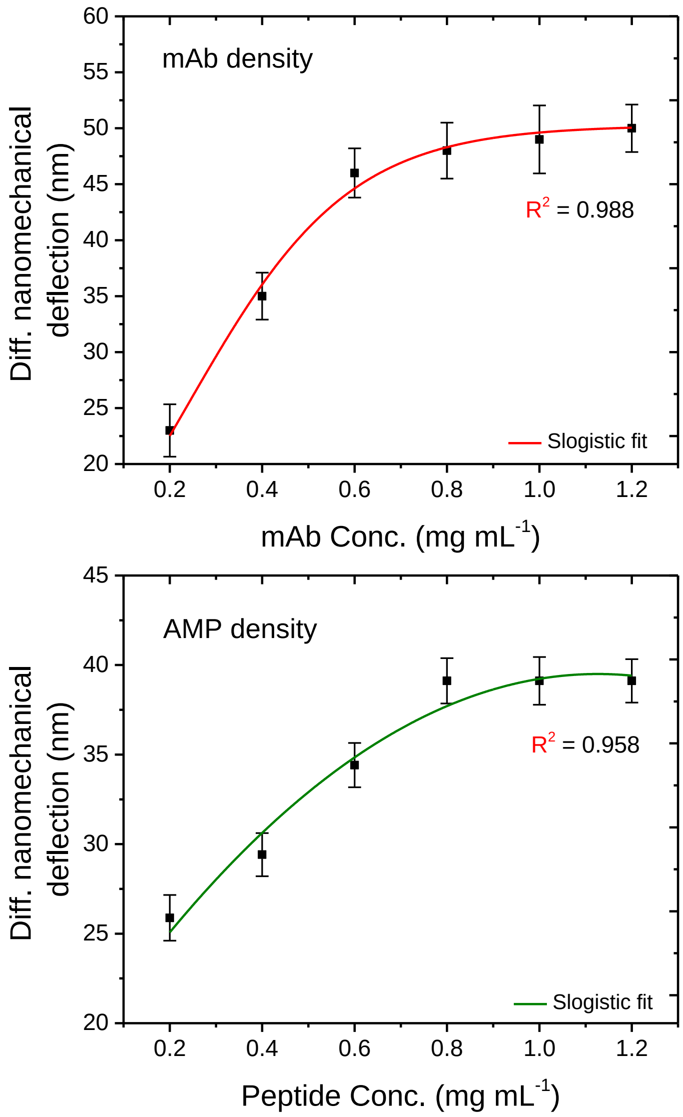

Supplementary Figure 3| adsorption density of the AMP and mAb on the BMC surface. The differential nanomechanical deflection induced is plotted against various ligand (AMP or mAb) concentrations in the sample (0.2 mg ml^{-1}, 0.4 mg ml^{-1}, 0.6 mg ml^{-1}, 0.8 mg ml^{-1}, 1 mg ml^{-1}, 1.2 mg ml^{-1}). The solid line represents the Slogistic calibration fit and error bars represent standard deviations (n = 5). The study suggested density saturation at 0.8 mg ml^{-1}, which was used subsequently for surface functionalization.

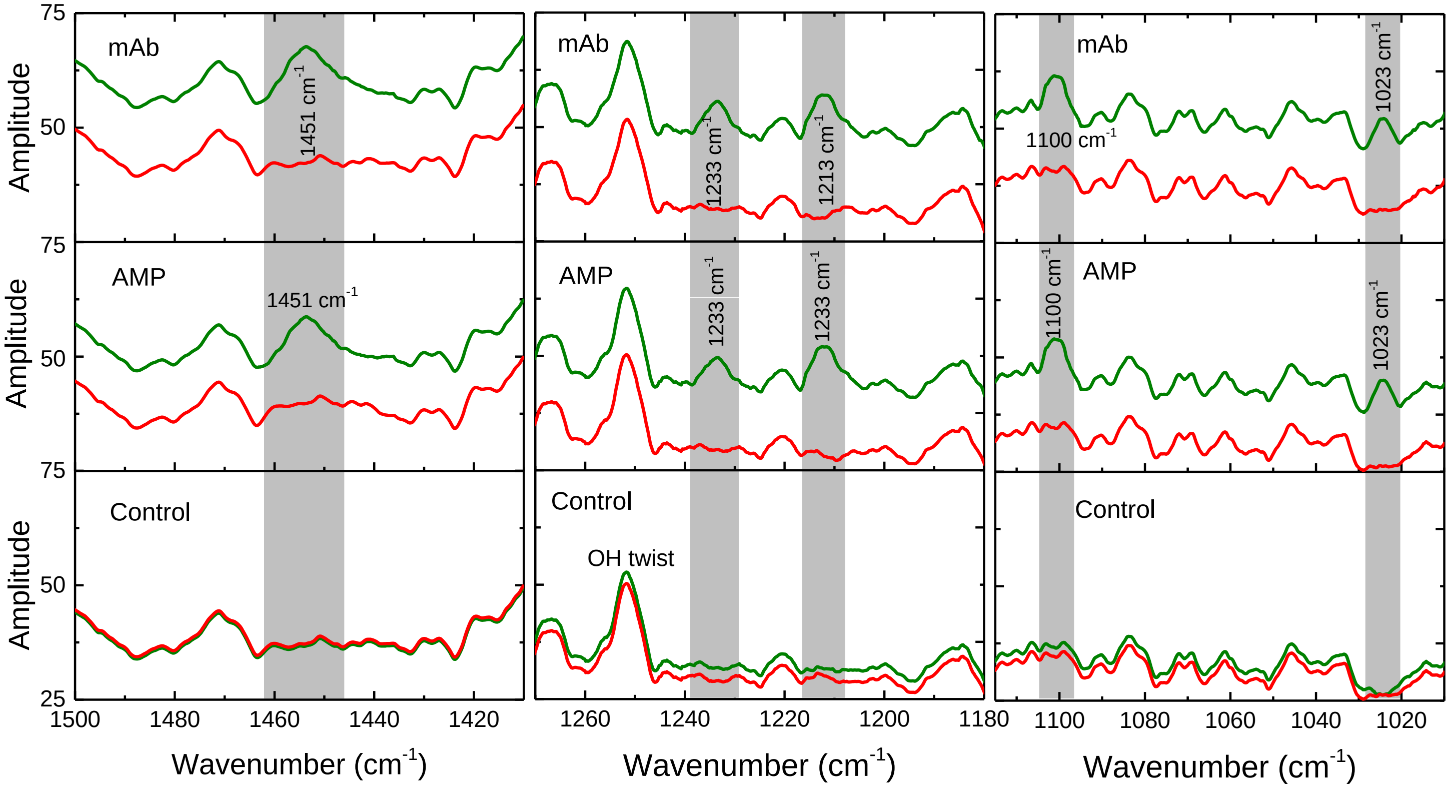

Supplementary Figure 4| IR-induced nanomechanical spectra show the fingerprint of L. monocytogenes at different wavelengths. As indicated, the spectra display linear nanomechanical signals associated with specific IR signatures of each adhesion layer added to the BMC. Adhesion or/ binding of bacteria (L. monocytogenes) to the immobilized ligands (mAb and AMP) show definite bacteria infrared absorption bands at different wavenumbers. In all spectra, red lines represent samples with no bacteria while the green lines represent the response to samples containing bacteria (100 cell /µL). While the mAb and AMP represent BMC coated with a monoclonal antibody and an antimicrobial peptide (Leucocin A), respectively, the control denotes a BMC coated with a negative peptide, which has no affinity to bacteria. The observed bacteria IR absorption bands, as specified above, 1451 cm^{-1}, 1233 cm^{-1}, 1213 cm^{-1}, 1100 cm^{-1} and 1023 cm^{-1} in the spectra correspond well to bacteria IR fingerprints illustrated previously by FTIR. Note that the presented spectra are smoothed (30%) short cut data of a wide range infrared spectrum.

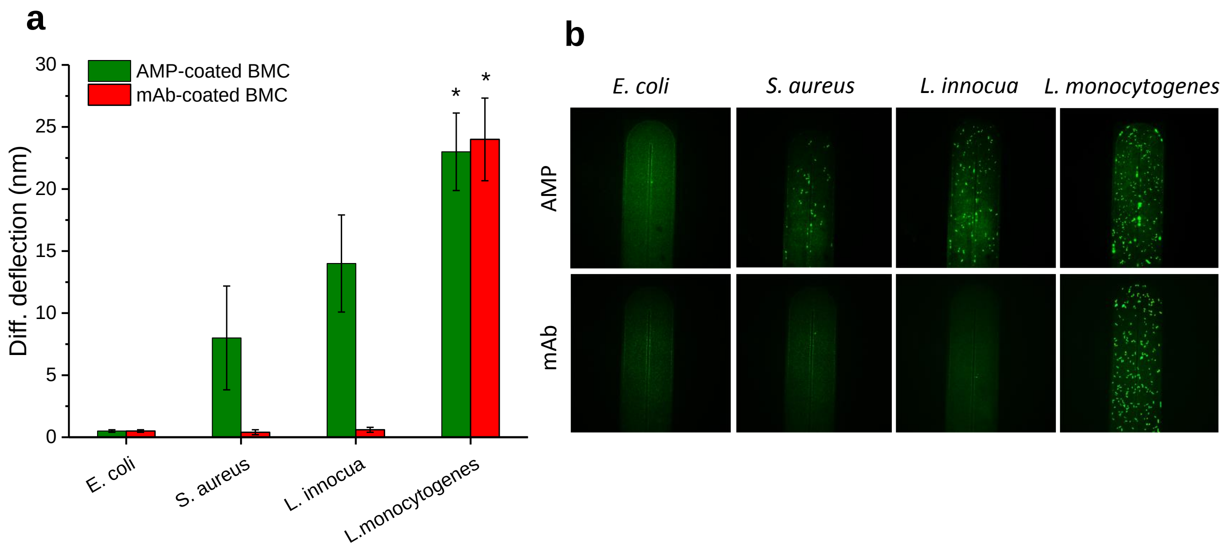

Supplementary Figure 5| nanomechanical deflection in response to various strains of bacteria. Charts show the differential cantilever deflection in response to various strains of bacteria. The results indicate statistical significant response to L. monocytogenes comparing to other strains with a P values > 0.05 (n = 5).

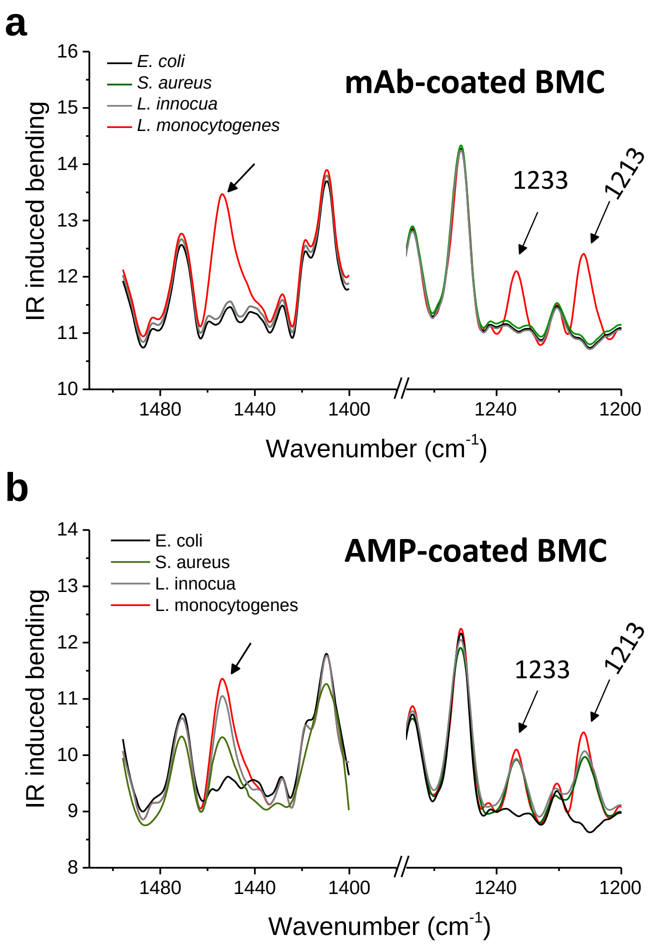

Supplementary Figure 6| The nanomechanical infrared spectra of captured bacteria inside the BMC sensors. Representative IR deviation analyzed spectra in the mid-IR region for bacteria strains as indicated, detected by exposure to a mAb-coated BMC sensor (a) or an AMP-coated BMC sensor. The IR absorption bands of the bacteria at ~ 1213 cm^{-1}, 1233 cm^{-1} and 1451 cm^{-1}, without extensive algorithmic preprocess (binning, smoothing and second deviation transformation) show the difference between strains of bacteria.

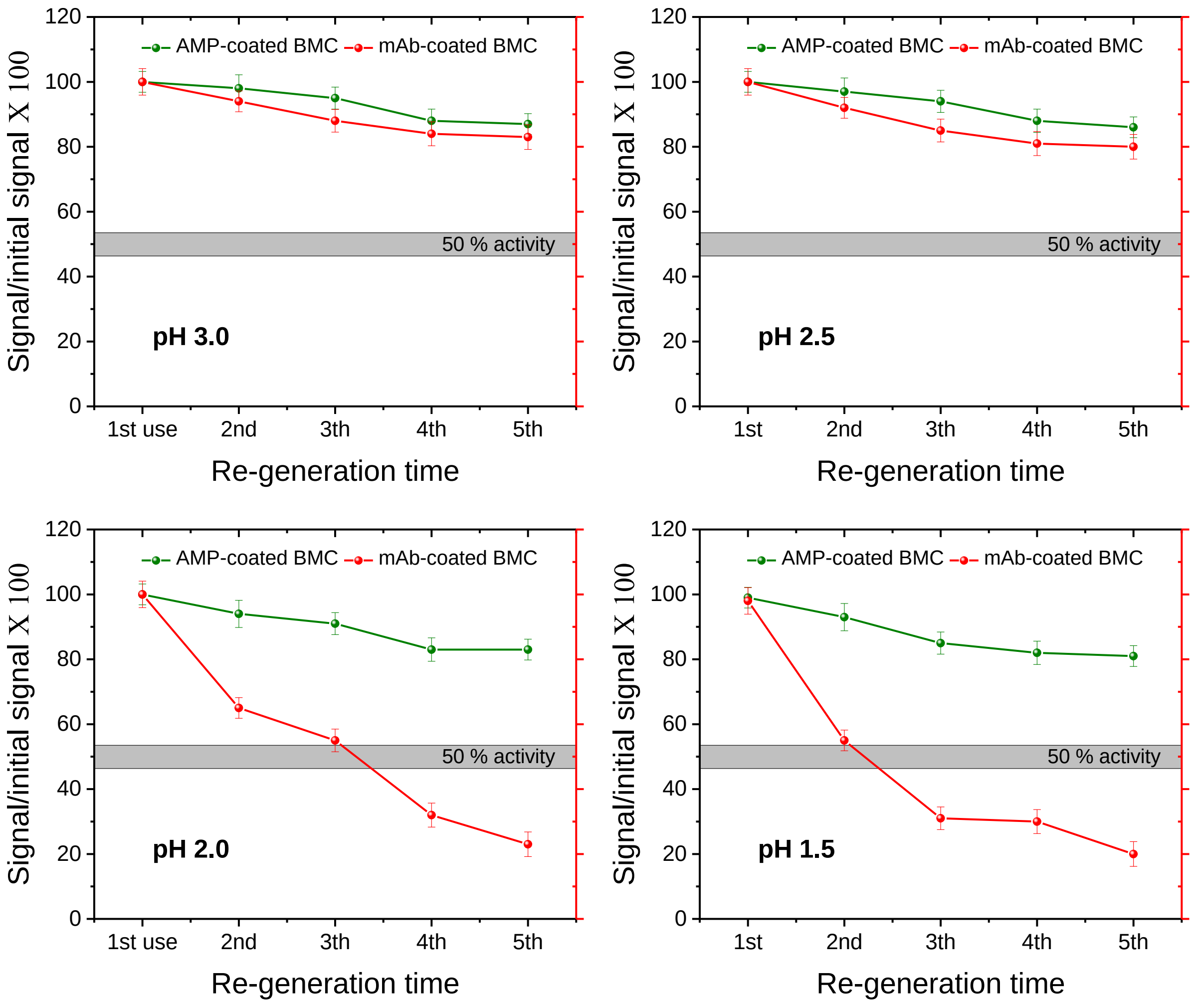

Supplementary Figure 7| BMC sensors re-usability. As indicated, performance of the BMC sensors, either the AMP-coated (green) or mAb-coated BMC (red) are shown after exposure to a repeated cycle of regeneration at various pH. Restored responses to analytes were presented as a percentage with respect to the sensors responses at their first time use. Each response is an average calculation of five replicates and error bars correspond to standard deviations.

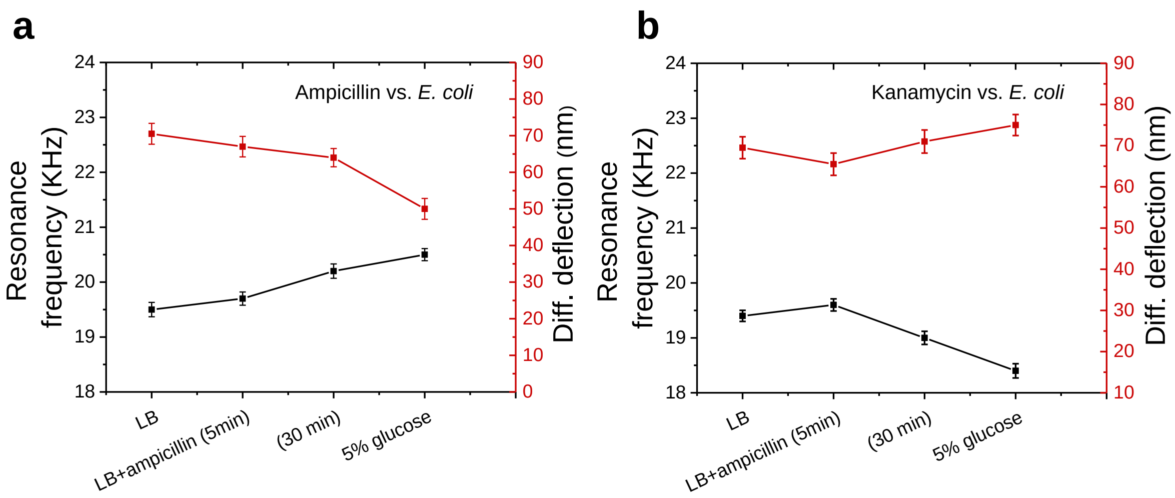

Supplementary Figure 8| Experiment shows the cantilever deflection and resonance frequency shift in response to bacteria metabolism. *E. coli* DH5α exposed to ampicillin (a) and kanamycin (b); the cantilever (deflection, frequency) measurements were performed as indicated, starting from bacteria in LB, adding antibiotics (10 µg ml^{-1}), 30 min after exposure to the antibiotics and 10 min after adding a 5% glucose solution. The results represent an average of 5 replicates performed at the same condition with error bars indicating standard deviation.

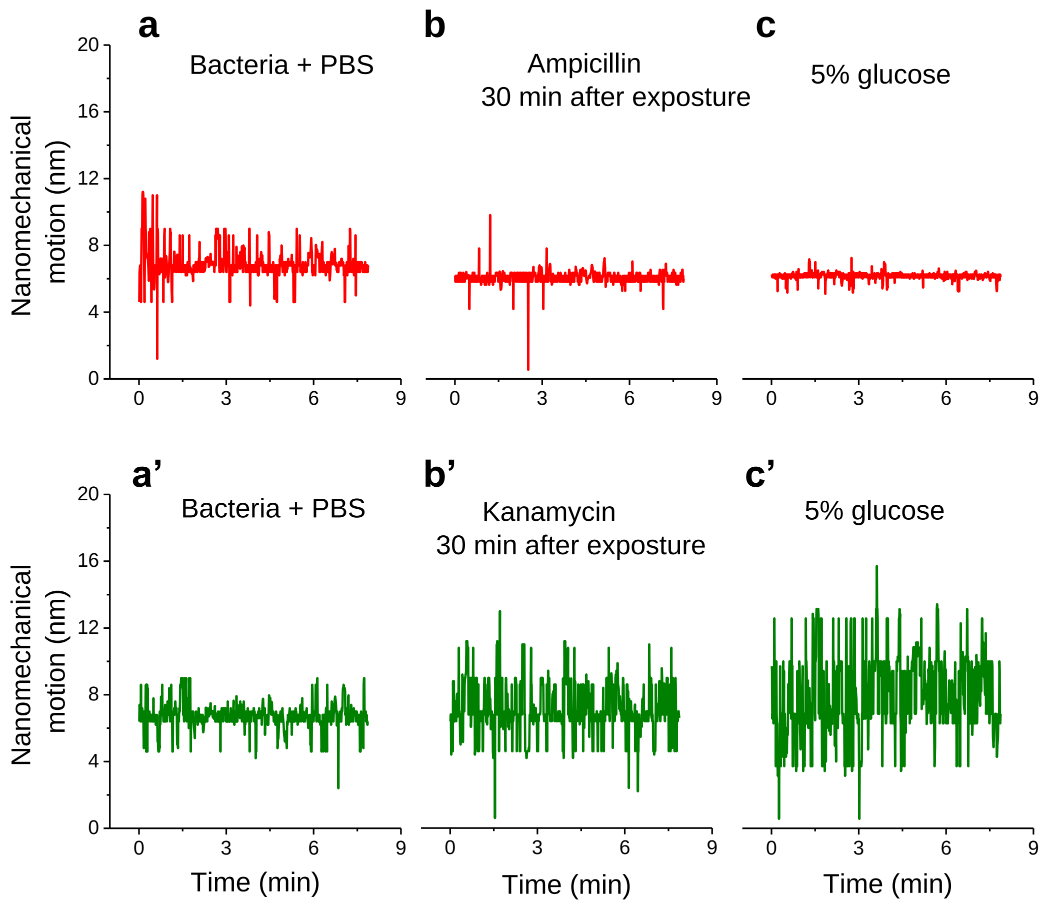

Supplementary Figure 9| Enhancement of nanomechanical BMC oscillation by bacteria (*E. coli*) metabolism. Nanomechanical fluctuation of bacteria exposed to ampicillin (upper panel) or to kanamycin (lower panel). **a** and **a'** are a result of bacteria in PBS, **b** and **b'** show a result of exposure to antibiotics, ampicillin or kenamycin, respectively (measurement after 30 min of exposure). **c** and **c'** shows the nanomechanical motion after exposure to 5% glucose solution by 10 min. The results suggested that *E. coli* is been killed by ampicillin but it resisted kanamycin. Removal of antibiotic to introduce 5% glucose to the bacteria enhanced the metabolism of the bacteria exposed to kanamycin to increase the nanomechanical fluctuation. While introducing ampicillin did not show any change of the cantilever fluctuation, indicating that bacteria is been killed.

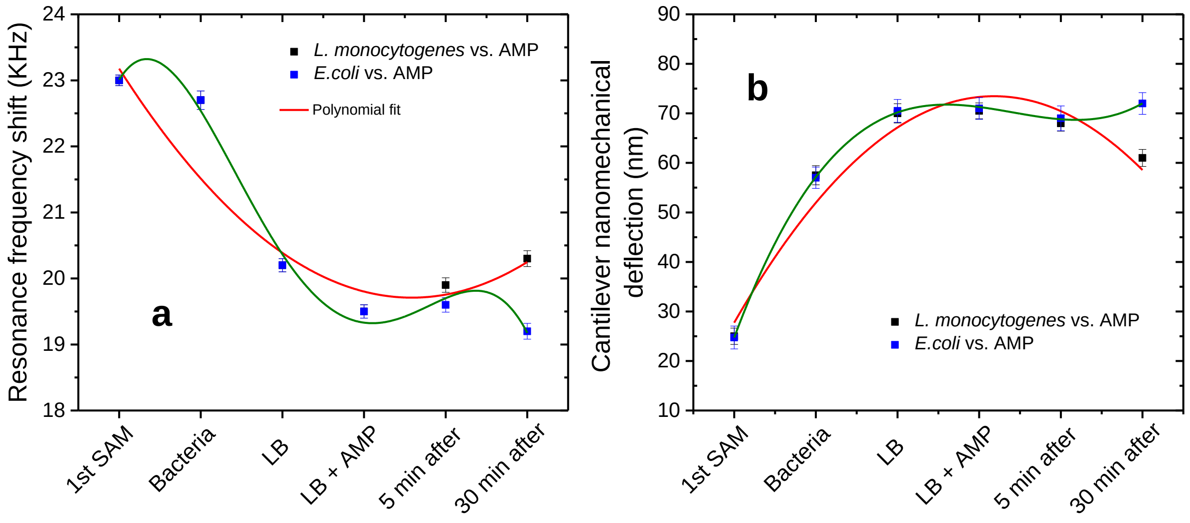

Supplementary Figure 10| Susceptibility of bacteria to the antimicrobial peptide Leucocin A. While the figure (**a**) shows resonance frequency shift as a result of bacteria (*L. monocytogenes and E. coli*) exposure to Leucocin A, figure (**b**) displays differential nanomechanical deflection as a result of exposure to Leucocin A at a concentration of 0.1 μg ml^{-1}. When Leucocin A was introduced into *L. monocytogenes* we see clear upward shift of the resonance frequency (**a red line**); however, when it was introduced into *E. coli* (**a green line**) a clear drop in the resonance frequency is observed after 30 min from the treatment. This suggests viability of the cells and its resistance to the antibacterial activity of Leucocin A. The experiment also describes the relation between the amplitude of BMC nanomechanical deflection and bacteria metabolic activity as indicated. During leucocin A injection, we observe a slight drop in the nanomechanical deflection after 5 min of leucocin A introduction. However, measurements at 30 min of exposure showed a decrease in the amplitude of deflection of *L. monocytogenes* sensor (**b red line**) and an increase in the amplitude of deflection for *E. coli* sensor (**b green line**). It is expected that Leucocin A has killed most of *L. monocytogenes* cells and deactivate its metabolic processes as what happen with ampicillin. The AMP however, has no effect on the *E. coli* cells; after few min from first shock the cells had recovered its metabolism. This causes an enhancement in the nanomechanical amplitude of deflection and decrease in the resonance frequency. Data is presented as a mean ± SD.

Supplementary Materials and Methods:

1. **Ligands and bacteria:**

In our bacterial detection study, the target strain of bacteria was *Listeria monocytogenes* (*L. monocytogenes*) – a facultative, food-borne pathogen with a mortality rate exceeds 20%.[1] Two *L. monocytogenes* targeting-molecules were employed, anti-*L. monocytogenes* monoclonal antibody (mAb) and *Listeria*-selective antimicrobial peptide (AMP). The mAb were purchased from MyBioSource, Inc. San Diego, California USA. The antimicrobial peptide (Leucocin A of class IIa bacteriocin), having a sequence of 37 amino acids (KYYGNGVHCTKSGCSVNWGE- -AFSAGVHRLANGGNGFW), was chemically synthesized using Fmoc-solid phase peptide synthesis (SPPS) as described in previous reports[2, 3]. We have chosen Leucocin A of class IIa bacteriocins because of its selectivity, potent activity against *L. monocytogenes*, ample stability and its applicable use in biosensing technologies as conveyed heretofore[3, 4]. A negative peptide (seq. CTKSGCSVNWGEAF), with no biological activity against bacteria[5] was used as a control for comparisons. Stocks of bacterial cells, *L. monocytogenes* ATCC 43256 (grown in TSBYE, 37 °C), *Listeria innocua ATCC* 33090 (grown in TSBYE, 37 °C), *Staphylococcus aureus ATCC* 13565 (grown in APT broth, 37 °C), and gram-negative *E.coli DH5α* (grown in LB media), were obtained from CanBiocin Edmonton Inc., subcultured, maintained and handled in a level II biosafety cabinet.

2. **Bimaterials microchannel cantilever (BMC):**

U-shaped microfluidic channel was made-up on top of a silicon nitride microcantilever having a dimension of 32 μm width, 600 μm lengths and a height of 3 μm (**Fig 1 in the main contents**). The cantilever was made bimetallic by coating the bottom side with a gold film (300 nm thickness). The inside part however, was kept as a thin layer of silicon nitride. The two openings

at the bottom of the lever, inlet and outlet, are attached to teflon tubes and adjusted to direct fluid flow (samples) in and out the microfluidic channel. The BMC chip settles in a holder made of polyether ether ketone (PEEK), connected to large tubes for fluid delivery. A polydimethylsiloxane (PDMS) seal is used to achieve a sealed contact between the PEEK holder and the BMC chip. An external-cavity quantum cascade lasers (QCLs) were concomitant the cantilever setup and used as a source of infrared (IR) light. When the BMC irradiated with a series of different IR wavelengths, the deflection is measured by reflecting a visible laser (635 nm) to a positive sensitive detector (PSD) attached to the system.

3. **BMC surface functionalization:**

The BMC channel consists of silicon nitride (Si_3N_4) was initially rinsed with piranha solution [30% H2O2/H2SO4, 1:3 (v/v)], chloroform, MQ-water and subjected to ambient atmosphere to ensure complete oxidation of the Si_3N_4 and formation of silicon dioxide assembles [*Note, piranha is extremely reactive, caution is strongly recommended*]. The formed silanol groups (Si - OH) were chemically attached to an ethanolamine linker ($NH_2C_2H_4OH$, Sigma Aldrich), which was further conjugated to the bacteria-targeting ligands (AMP or/ mAb). In details, ethanolamine hydrochloride (300 mg) was dissolved in DMSO (1 mL) under gentle heating at ~70 C in a crystallization dish. It was allowed then to cool down and degassed in a desiccator at aspirator vacuum for 30 min. A solution of ~100 μL was subsequent passed through the BMC several times during the day and the chip was sealed by Teflon and incubated overnight in the solution to ensure a complete coupling. Next, the chip was rinsed with ethanol and dried under stream of nitrogen gas. Either AMP /or mAb solution (0.8 mg mL^{-1}), activated by NHS/EDC for 10 min, was injected to the BMC chip five times, ~50 μL once every 2 hr to ensure a widespread

assembling. The solution was also kept overnight in the microchannel to certify the functionalization (**Fig S1)**. Prior experiments, the BMC were washed with MQ-water, ethanol and dried under stream of nitrogen gas.

4. Surface characterization and ligands density measurements:

In the initial experiments the resonance frequency and the infrared-induced nanomechanical deflection of the BMC functionalized with self-assembled monolayers (SAMs) of ethanolamine (1st SAM), and AMP or/ anti-listeria monoclonal antibody (mAb) as 2^{nd}SAM were collected individually. Using a Quantum Cascade Laser (QCL) (MIRCat, Day Light Solutions), the BMC was irradiated with tunable IR light at different wavenumber spans, QCL1 (from 1615 cm^{-1} – 1365 cm^{-1}), QCL2 (1365 cm^{-1} – 1170 cm^{-1}) and QCL3 (1700 cm^{-1} – 999 cm^{-1}) to achieve a longer range of IR signatures. The average IR nanomechanical spectrum (average of the amplitude) represents the averaged measurements of five independent experiments performed under same conditions. The average amplitudes were plotted against wavenumbers cm^{-1} to present the nanomechanical deflection and the IR fingerprint spectra of the delivered samples.

We examined the surface functionalization process by observing the changes in the nanomechanical IR readings, resonance frequency and nanodeflection of the cantilever compared to the background spectrum of silicon nitride. The 1st SAM (ethanolamine layer) was detected by appearance of a distinctive absorption peak at ~1600 cm^{-1}, suggesting a primary amine absorption peak (**Fig S2a**). The peak however, nearly vanished subsequent loading of the 2nd SAM (AMP or/mAb adlayer), signifying the success of the peptide conjugation to the ethanolamine layer and indicating a constant adlayer formation (**Fig S2a**). The adlayer of the AMP and/or mAb was also defined by the appearance of a strong absorption peak at 1533 cm^{-1},

which corresponds well to the amide II absorption band[6, 7]. Furthermore, the differential analysis of amplitudes of nanomechanical deflections (**Fig S2b**) and the resonance frequency shifts (**Fic S2c**) showed the differences in the mass densities of the two adsorbed layers (1st and 2nd SAMs), and indicated the attainment of the sensor surface activation.

To ensure a high surface density of the immobilized ligands on the surface of the BMC chip, we performed preliminary tests, where diluted samples of antimicrobial peptide (Leucocin A) and mAb (0.2, 0.4, 0.6, 0.8, and 1 mg ml^{-1}) were introduced into the BMC sensor and subjected to nanomechanical readings. The nanomechanical cantilever bending was computed and results were presented as differential deflection against concentration of the peptide in the samples (**Fig S3**). Results suggested that a concentration of 0.8 mg mL^{-1} is an optimum to achieve maximum surface density of both AMP and mAb. Based on the results, we used the highest concentration of 0.8 mg mL^{-1} for immobilization of the ligands in the BMC microchannel.

5. **BMC measurements; bacterial detection/sensitivity and selectivity**:

BMC fabrication, instrumentation for data acquisition and software for data analysis are described in our previous report[8]. Here, by passing the fluid through the channel, the device is not only detecting the change in the total mass density of the cantilever, but it also identifies the molecular fingerprint of the present molecules in the sample by providing the nanomechanical IR spectra of the entire delivery. Initially, the measurements was performed to characterize the surface of the channel and to endorse the surface functionalization processes, as illustrated above.

In the bacterial detection experiments, artificially contaminated samples with *L. monocytogenes* at 10^3 cfu mL^{-1} (100 cells /100 µl were conceded through the BMC sensors, incubated and subjected to the nanomechanical readings. Three different readings, IR signatures, magnitude of nanomechanical cantilever deflections and resonance frequency shifts were measured. To estimate the device sensitivity and limit of detection, diluted samples with bacterial cells suspended in water at a range of 10^3 – 10^6 cfu ml^{-1}were subjected to the sensors readings. Various strains of bacteria were also exposed to the BMC sensors in order to determine the sensors selectivity. Each experiment was repeated at least five times under same conditions and at different time sets. Signals of the readings were plotted with respect to the wavenumber of IR light that generates nanomechanical IR spectra of the analytes inside the BMC. The IR spectral features are often overlapped. Thus, some data preprocessing were performed to analyze the data, such as binning, smoothing, and second derivative transformation analysis. Binning reduces the number of data points in a spectrum, smoothing eliminates noise by averaging neighboring data points. Second-derivative transformation separates overlapping absorption bands and removes baseline offsets.

6. **<u>Confocal microscopy:</u>**

A Stock solution of the CyQUANT dye (a green color probe) was made by following the manufacture protocol. Briefly, CyQUANT probe reagent (0.8 μL) was dissolved in HBSS buffer (200 μL) and stored in dark condition at 4 °C. Bacterial cells (*L. monocytogenes, or L. innocua, or S. aureus, or E. coli DH5α*) were pulled from the culture by centrifugation and re-suspended in fresh 1× PBS solution. The supernatant was eliminated and the bacterial cells at a concentration of 10^6 cfu ml^{-1} were incubated with CyQUANT solution (100 μL) for ~30 min at

37 °C. The cells were pelleted by centrifugation again and resuspended in fresh 1 × PBS buffer. Samples of stained bacterial cells were then introduced into the AMP-coated BMC sensor and the mAb-coated BMC, independently for ~30 min. A gentle wash of the BMC sensors with 1 × PBS was performed prior any microscopic examination. The captured bacterial cells were examined using a Quorum WaveFX spinning disk confocal microscopy (Quorum Technologies Inc., Guelph, Canada) through a magnification20×/1.4. All captured images were recorded using a Quorum digital camera and were analyzed using a velocity three-dimensional image analysis software.

7. Sensor re-usability:

Effective regeneration is a key for successful sensor assays. Therefore, a valuable investment would be establishing a suitable re-generation condition that allows a number of recycling with maintaining a sufficient activity and efficient performance. The BMC chip of both, AMP-coated and mAb-coated sensors, was simply re-generated via two steps. First, the sensors were vigorously washed with a regeneration solution of 10 mM glycine-HCl at pH (3.0, 2.5, 2.0 or 1.5), independently. The microfluidic channel was then rinsed with 70% ethanol in order to remove all adsorbed materials and making the functionalized BMC sensor accessible again for further detection assays. Performance of the re-generated BMC chips was evaluated against *L.* monocytogenes at 100 cells per 100 μL. The nanomechanical deflection was measured at the indicated bacteria infrared signature of both regenerated sensors (AMP-coated or mAb-coated BMC). The average calculated values were obtained as percentage of responses with respect to the initial deflection values obtained at the first time use. The condition of suitable regeneration was determined by using different pH values as indicated. **Fig S6** shows the sensors performance after BMC regeneration. It turns out that regeneration of the AMP-coated BMC at all conditions

tested restore the immobilized receptors response to the analyte to a constant level, which is 80% or more with best regeneration achieved at pH 2.5 and 3.0. Accordingly, the regeneration can be performed confidently at pH 3.0 or 2.5. In contrast to the AMP sensor, the repeated usage of the mAb-coated BMC sensor after its regenerations at harsh environment (pH 2.0 and 1.5) had resulted in dramatic loss of its binding activity to more than 50%, which indicate that the immobilized mAb may undergo unfolding and denaturation (**Fig S6**). However, the sensor was sufficiently stable at milder conditions (pH 2.5 and 3.0) where a restored response reaches ~80%. The results suggest that mAb-coated BMC can be regenerated at pH 2.5 – 3.0 (or possibly higher), but it loses significant activity at lower pH. Steps for BMC sensor regeneration may need further optimizations using further reagents since milder conditions showed to preserve the sensor performance.

8. <u>**Bacteria drug resistance**</u>

All chemicals, culture media, reagents and antibiotics (ampicillin and kanamycin), with analytical grade, were obtained from Sigma-Aldrich. The ampicillin is a β-lactam containing antibiotics, penicillin alike, that act by inhibition of bacterial cell-wall synthesis through its interference with the peptidoglycan biosynthesis. The kanamycin; on the other hand, is an aminoglycoside subtype that kills bacteria by causing a membrane-damage and inhibiting DNA and RNA synthesis. *E. coli DH5*α is a well-known strain with its sensitivity to ampicillin and its resistance to kanamycin. The AMP (Leucocin A) was also applied in this study in order to verify applicability of the sensor to detect various drug-resistances and to explore response of the bacteria to antibiotics and antimicrobial peptides. Leucocin A is very unique class IIa bacteriocin peptide, with very strong activity against *L. monocytogenes.* The peptide acts by targeting specific membrane allocated receptor found on the surface membrane of bacterial cells that is

known as mannose phosphotransferase[5]. Interestingly, some bacterial cells express this receptor and others do not; some cells have higher expression level of this receptor than others and some cells develop resistance gene to modify this targeted receptor. In order to identify bacterial resistance to this AMP, we have used two different strains of bacteria, *E. coli DH5α* and *L. monocytogenes*. While *L. monocytogenes* is very sensitive to Leucocin A, DH5α-strain is unsusceptible to it [5].

9. Bacteria preparations

As described previously, frozen stocks of bacteria, stored at -80°C in glycerol-supplemented media, were initially streaked in agar growth media and few bacterial colonies were collected afterward and incubated overnight at 37°C in 1 ml of broth media (LB for *E. coli* DH5 and TSBYE for *L. monocytogenes* 43256). After incubation, the bacterial culture was centrifuged; bacteria were precipitated and re-suspended in a phosphate buffered saline – pH 7.4.

10. BMC sensor preparation, calibration and detection of bacterial-drug resistance

Our home-made silicon nitride microchannel cantilevers coated from bottom with a thin film of gold layer (300 nm) and having dimensions of 32 μm width, 600 μm lengths and a microchannel height of 3 μm were embedded on it. Initially, the BMC was treated with (3-aminopropyl) triethoxysilane (APTES) – a linker molecule that promotes adherence of bacterial cells to the cantilever surface. The linker provides loose attachment of the cells to the cantilever surface without affecting its metabolic and viable activities [9]. Specifically, the BMC was subjected to a 0.2% solution of APTES in MQ water for approximately 3-5 min and then rinsed with ultrapure water. The BMC sensor was introduced into the sensor chamber for analysis and calibration. The

calibration was performed by injecting buffer solution free from bacteria, and taking its nanomechanical reading as a baseline for measuring the subsequent experiments. Bacteria cells either *E. coli*, in case of (ampicillin and kanamycin or *Listeria monocytogenes*, in case of the AMP Leucocin A, were diluted at 10^{-5} and introduced into the BMC. The cells were left to incubate for ~10 min at room temperature and were then rinsed gently with PBS to remove any floating bacterial cells. Standard LB media or LB media containing antibiotics were injecting individually to the BMC sensor and data of the resonance frequency, cantilever deflection and IR signatures were measured simultaneously after each step. The measurements were performed at 5 min from the injection and after 30 min from the injection. The measurement was performed also after the antibiotics were removed and re-introduced LB media again. In addition, in order to enhance the metabolism of the bacteria, we introduced 5% glucose solution to bacteria after exposure to antibiotics and measured the sensor response 10 min later. The experiments are intended to elaborate the viability of the cells and its susceptibility to the treatments. Due to overlapping, the IR spectral preprocessing such as binning, smoothing, and second derivative transformation analysis were performed to analyze the data. Binning reduces the number of data points in a spectrum, smoothing eliminates noise by averaging neighboring data points. Second-derivative transformation separates overlapping absorption bands and removes baseline offsets. In addition, in order to differentiate intact from dead bacteria. IR Multivariate analysis, analogous to principal component analysis (PSA), was performed to differentiation life from dead bacteria. The analysis involved applying a stepwise variable selection to decrease the multidimensionality of the data into its most significant scores as described previously.

11. Bacteria viability assay (Microscopy)

The viability of bacterial cells attached to the inner walls of the cantilever was evaluated using a live/dead Bacterial Viability Kit (Life Technologies Inc., Burlington, ON, Canada). Live/dead bacterial viability stains includes CyQUANT green and propidium iodide (PI). The two dyes were prepared separately by dilution in MQ-water (1:10) and then mixed together in equivalent ratio (1:1 vol/vol). The mixed live/dead solution (~ 10 µL) was introduced into BMC contains bacteria had exposed either to ampicillin or kanamycin. The BMC left for 10 min in darkness at room temperature prior to analysis. The captured bacteria were examined using a Quorum WaveFX spinning disk confocal microscopy (Quorum Technologies Inc., Guelph, Canada) through a magnification20×/1.4. All captured images were recorded using a Quorum digital camera and were analyzed using a velocity three-dimensional image analysis software.

12. Statistical analysis:

All nanomechanical measurements were averaged and each experiment was performed at least five times. Data are presented as mean ± SD throughout the manuscript. The statistical difference was tested either using the unpaired t-test or the one way ANOVA test. In all statistical analysis the significance level (P value) was sat at as 0.05.

Supplementary References

1. CDC Incidence and Trends of Infection with Pathogens Transmitted Commonly Through Food — Foodborne Diseases Active Surveillance Network,10 U.S. Sites, 2006–2013. *MMWR Morb Mortal Wkly Rep* **63**, 328-332 (2014).

2. Etayash, H., Norman, L., Thundat, T., Stiles, M. & Kaur, K. Surface-Conjugated Antimicrobial Peptide Leucocin A Displays High Binding to Pathogenic Gram-Positive Bacteria. *ACS Appl Mater Interfaces* **6**, 1131-1138 (2014).

3. Etayash, H., Norman, L., Thundat, T. & Kaur, K. Peptide-Bacteria Interactions using Engineered Surface-Immobilized Peptides from Class IIa Bacteriocins. *Langmuir* **29**, 4048-4056 (2013).

4. Etayash, H., Jiang, K., Thundat, T. & Kaur, K. Impedimetric Detection of Pathogenic Gram-Positive Bacteria Using an Antimicrobial Peptide from Class IIa Bacteriocins. *Anal Chem* **86**, 1693-1700 (2014).

5. Hashem Etayash, S.A., Ramana Dangeti and Kamaljit Kaur Peptide Bacteriocins - Structure Activity Relationships *Curr Top Med Chem* **16**, 220 - 241 (2015).

6. Kong, J. & Yu, S. Fourier transform infrared spectroscopic analysis of protein secondary structures. *Acta Biochim Biophys Sin* **39**, 549-559 (2007).

7. Costa, F., Carvalho, I.F., Montelaro, R.C., Gomes, P. & Martins, M.C. Covalent immobilization of antimicrobial peptides (AMPs) onto biomaterial surfaces. *Acta Biomater* **7**, 1431-1440 (2011).

8. Faheem Khan, M. et al. Nanomechanical identification of liquid reagents in a microfluidic channel. *Lab on a Chip* **14**, 1302-1307 (2014).

9. LongoG et al. Rapid detection of bacterial resistance to antibiotics using AFM cantilevers as nanomechanical sensors. *Nat Nano* **8**, 522-526 (2013).
